# Menstrual hygiene management among women and adolescent girls in the aftermath of the earthquake in Nepal

**DOI:** 10.1186/s12905-018-0527-y

**Published:** 2018-02-02

**Authors:** Shyam Sundar Budhathoki, Meika Bhattachan, Enrique Castro-Sánchez, Reshu Agrawal Sagtani, Rajan Bikram Rayamajhi, Pramila Rai, Gaurav Sharma

**Affiliations:** 1 School of Public Health & Community Medicine, B P Koirala Institute of Health Sciences, Dharan, Nepal; 2 NIHR Health Protection Research Unit in Healthcare Associated Infection and Antimicrobial Resistance at Imperial College London, London, UK; 3 Patan Academy of Health Sciences, Kathmandu, Nepal; 4 Freelance Researcher, Kathmandu, Nepal; 5 Department of Infectious Disease Epidemiology and Maternal, Adolescent, Reproductive and Child health (MARCH) Centre, London School of Hygiene and Tropical Medicine, London, UK

**Keywords:** Menstrual hygiene, Nepal earthquake, Sexual and reproductive health, women’s health

## Abstract

**Background:**

Menstrual hygiene management (MHM) is an essential aspect of hygiene for women and adolescent girls between menarche and menopause. Despite being an important issue concerning women and girls in the menstruating age group MHM is often overlooked in post-disaster responses. Further, there is limited evidence of menstrual hygiene management in humanitarian settings. This study aims to describe the experiences and perceptions of women and adolescent girls on menstrual hygiene management in post-earthquake Nepal.

**Methods:**

A mixed methods study was carried out among the earthquake affected women and adolescent girls in three villages of Sindhupalchowk district of Nepal. Data was collected using a semi-structured questionnaire that captured experiences and perceptions of respondents on menstrual hygiene management in the aftermath of the Nepal earthquake. Quantitative data were triangulated with in-depth interview regarding respondent’s personal experiences of menstrual hygiene management.

**Results:**

Menstrual hygiene was rated as the sixth highest overall need and perceived as an immediate need by 18.8% of the respondents. There were 42.8% women & girls who menstruated within first week of the earthquake. Reusable sanitary cloth were used by about 66.7% of the respondents before the earthquake and remained a popular method (76.1%) post-earthquake. None of the respondents reported receiving menstrual adsorbents as relief materials in the first month following the earthquake. Disposable pads (77.8%) were preferred by respondents as they were perceived to be clean and convenient to use. Most respondents (73.5%) felt that reusable sanitary pads were a sustainable choice. Women who were in the age group of 15-34 years (OR = 3.14; CI = (1.07-9.20), did not go to school (OR = 9.68; CI = 2.16-43.33), married (OR = 2.99; CI = 1.22-7.31) and previously used reusable sanitary cloth (OR = 5.82; CI = 2.33-14.55) were more likely to use the reusable sanitary cloth.

**Conclusions:**

In the immediate aftermath of the earthquake, women and girls completely depended on the use of locally available resources as adsorbents during menstruation. Immediate relief activities by humanitarian agencies, lacked MHM activities. Understanding the previous practice and using local resources, the reusable sanitary cloth is a way to address the menstrual hygiene needs in the post-disaster situations in Nepal.

## Background

Menstruation is a naturally occurring physiological phenomenon in adolescent girls and pre-menopausal women [1]. Menstrual Hygiene Management (MHM) is defined as *‘Women and adolescent girls using a clean menstrual management material to absorb or collect blood that can be changed in privacy as often as necessary for the duration of the menstruation period, using soap and water for washing the body as required, and having access to facilities to dispose of used menstrual management materials’*. Menstruation necessitates the availability of material resources to absorb or collect menstrual blood, facilitate personal hygiene and dispose of waste, ideally with adequate privacy [2]. Women and girls in low income settings have low awareness on hygienic practices and lack culturally appropriate materials for menstrual hygiene management (MHM) practices [3–5]. Menstruation and associated activities are surrounded by silence, shame and social taboos that are further manifested in social practices that restrict mobility, freedom and access to normal activities in India and Nepal. For instance, drinking milk, preparing food, interacting with people or refraining from performing religious rituals are restrictions found in many cultures [5–9].

The materials used as adsorbents during menstruation in low income countries including Nepal, vary from reusable towels (cloth torn from dresses of women and cotton fabric) to commercially available disposable sanitary pads [6, 10–15]. Practical, sustainable and culturally acceptable methods are recommended for addressing the menstrual hygiene needs of women in low income countries [4, 16]. Types of adsorbents used, hygienic practices and cultural restrictions during menstruation are associated with negative clinical and psychosocial outcomes including reproductive and urinary tract infections, anemia, school absenteeism, and social isolation [1, 6, 15, 17, 18].

Young adolescent girls tend to be less prepared for MHM and suffer from anxiety, apprehensions, fear and shame during their menses [12, 19]. In addition, pre-existing social taboos and cultural restraints during menstruation mean that managing menstruation is a greater challenge during disasters [20–22]. Further, there is limited access to reproductive health services and safe menstrual hygiene materials during disasters [23]. Menstrual hygiene needs are not only specific and pressing to women and girls in reproductive age but also require access to same management of the menstrual period, a basic reproductive health right. In emergencies, the usual lifestyles of affected individuals change and they are confronted with additional stress that could worsen their physical and psychological well-being. Provision of fundamental human requirements such as shelter, food, clean water and medicines is prioritised, however other needs such as safe menstrual hygiene management that can have profound psychosocial impact if unmet are often neglected [22, 24].

On 25^th^April 2015, Nepal was affected by a 7.8 Richter scale magnitude earthquake killing almost 9000 people and displaced 3.5 million people [25]. Compared to men, more women survived [26] and although emergency relief and rescue operations swiftly began addressing essential humanitarian needs, there seemed to be a lack of attention to menstrual hygiene needs of adolescent girls and women [13, 27]. Although increasingly considered, menstrual hygiene continues to be overlooked during disasters, even in community level responses [22, 28, 29]. Nepal is frequently affected by disasters, both natural as well as manmade leading to displacement of the population into temporary shelters [25, 30].

Menstrual hygiene is a need of menstruating women & girls. Although, there are some studies on menstrual hygiene management in Nepal, there is limited evidence on menstrual hygiene management especially in the aftermath of the earthquake in Nepal [12, 13, 21, 31]. Therefore, we conducted this study to explore the perceptions and the lived experience of women and young girls regarding menstrual hygiene management in the aftermath of the earthquake in Nepal.

## Methods

### Study design& settings

This study was conducted amongst 117 women and adolescent girls in three villages of Sindhupalchowk district of Nepal. The selected district was chosen since it was one of the most earthquake affected district [26]. The data collection was conducted from 16th August until 29th August 2015.

### Sampling strategy and sample size

We purposely selected Chautara (the main hub of Sindupalchowk district) [32] and 2 other villages adjacent to Chautara (Kubhinde and Pipaldanda) for our study. According to the 2011 census of Sindhupalchowk district, the population of girls and women in the age group of 15 - 49 years in the study areas was 3484 (Chautara = 1759; Kubhinde = 784; & Pipaldanda = 941). As the earthquake affected 65% of the population of the district, the estimated number of women and girls eligible to participate in the study would be 2316. We planned to interview 5% of this target population with 10% amplification for possibility of non-response errors. Thus, the estimated final sample size was 127.

### Participants

A purposive sampling technique was employed to invite 127 women and adolescent girls living in temporary shelters set up in the district to participate in the study. Adolescents who had not had menarche and menopausal women were excluded from the study. The interviews were scheduled at the time of the day when there were no relief materials being distributed in the areas and the women and girls were free to participate.

### Data collection and analysis

Quantitative data was collected using a semi-structured questionnaire comprising of socio-demographic characteristics, menstrual practices prior to the earthquake, perceived needs and menstrual hygiene practices during the post-earthquake period and preferred adsorbents for MHM during the aftermath of the earthquake. Face-to-face interview conducted by female interviewers are graduate medical and nursing professionals who were given 3-4 h orientation on both quantitative and qualitative questions before the data collection by the research team. The questionnaire was finalised in English and then translated into Nepali before the data collection using the translation-back translation method. The interviews were conducted in Nepali language. Pretesting of the questionnaire was conducted with women and adolescent girls residing in temporary settlement areas of Kathmandu (*n* = 20) to ensure that questions were socio-culturally acceptable and completely answered our research question.

Additionally, qualitative data was collected from a number of respondents (*n* = 5) who had their menses on the day or within the first week of the earthquake. The women and girls were invited to participate in a brief in-depth interview, which lasted for 10-15 min, including open questions on menstruation experience and its management (‘*Could you share your experience on your first menstruation after the earthquake?’* and *‘Could you elaborate on the management of your menstruation’*). A female interviewer recorded the interview and took field notes. The interview took place in Nepali language. The recording was later transcribed as verbatim.

The quantitative data was initially entered in Microsoft Excel and later analysed using the SPSS Statistics for Windows, Version 21.0. Armonk, NY: IBM Corp. The qualitative material was analysed by the researchers using thematic analysis [33]. An initial thematic framework was developed using a deductive-inductive approach. The framework was refined and used to analyse the remaining interviews. Transcripts were examined line-by-line and coded using thematic analysis. Key categories and themes were identified and iteratively compared within the framework to elicit further emerging codes. Thematic saturation was considered once the analysis of data offered no new information and a redundancy of categories.

### Ethical considerations

Ethical approval for this research was obtained from the Institutional Review Committee of B P Koirala Institute of Health Sciences (Ref: IRC/634/015), Dharan, Nepal. Prior to the start of the data collection, researchers met with community leaders and outlined the purpose and objectives of the study. The research team were external to the ongoing relief operations and had no influence on their logistics or other conflict of interests. After receiving approval from the community leaders and with the support of the local women’s group representative, suitable participants were identified. Literate women were given an information sheet that provided details on the research objectives, expected role of the respondents and the voluntary nature of participation. They were informed that their decision to participate or decline participation would not affect any benefits or services received by them. Written informed consent was obtained from all literate participants. For women unable to read, the information sheet was read out and the research objectives, expected roles of participants and voluntary nature of participation was explained in the presence of a literate female elder. A thumb print was obtained and this was countersigned by another witness. For respondents between 15 and 17 years, an assent form was used along with the permission of a parent/guardian to participate in the study.

## Results

The research team identified 127 eligible individuals, of whom 117 agreed to participate (response rate 92.1%). Table 1 presents sociodemographic characteristics of the participants. Almost 65% (77) of the respondents had some schooling, with 10% (11) attaining a university level education. More than 70% (84) of respondents were married. About 67% of respondents reported using a reusable cloth as a sanitary adsorbent during menstruation regularly before the earthquake. More than 80% (102) of respondents changed adsorbents two or more times daily during menstruation (Table 1).

**Table 1:** Sociodemographic characteristics of study participants (*n* = 117)

Variables	No (%)
Age	
15-24 years	42 (35.9%)
25-34 years	40 (34.2%)
35-49 years	35 (29.9%)
Education	
No formal schooling	40 (34.2%)
Primary School	6 (5.1%)
Secondary School	60 (51.3%)
University	11 (9.4%)
Marital Status	
Unmarried	33 (28.2%)
Married	84 (71.8%)
Adsorbents type used before earthquake	
Disposable Pads	39 (33.3%)
Reusable Cloth	78 (66.7%)
Frequency of adsorbent changes/day during menstruation before earthquake	
Once	15 (12.8%)
Twice	54 (46.2%)
Thrice or more	48 (41.0%)
Start of menses in relation to earthquake on 25 April 2015	
Menstruating at the time.	3 (2.6%)
In first week, post- earthquake	47 (40.2%)
In second week, post- earthquake	34 (29.1%)
In third week, post- earthquake	24 (20.5%)
In fourth week, post- earthquake	9 (7.7%)

When respondents were asked to recall up to five immediate needs following the earthquake; food (97%), shelter (75.2%), water (63.2%), clothes (47.0%) and information about family members/relatives (29.9%) were the five highest ranked needs. Menstrual hygiene was the sixth highest perceived need, listed by 18.8% of the respondents (Table 2).

**Table 2:** Immediate post-earthquake needs as perceived by study respondents

Variables	No (%)
Perceived need*	
Food	114 (97.4%)
Shelter	88 (75.2%)
Water (Drinking & Washing)	74 (63.2%)
Clothes	55 (47.0%)
Information about Family Members/relatives	35 (29.9%)
Adsorbents for Menstrual Hygiene	22 (18.8%)
Mobiles	17 (14.5%)
Medicine/First aid	13 (11.1%)
Safe place	12 (10.3%)
Soap	8 (6.8%)
Cattle	7 (6.0%)

*Multiple responses

Within the first month of the earthquake, none of the respondents reported having received any menstrual hygiene adsorbents within the distributed relief materials. Soap was received by 50.4% of women and girls. Availability of a safe space with adequate privacy for changing of adsorbents was reported by 42.7% of respondents. About 12% of women perceived the need for adsorbents required for menstrual hygiene management on the same day just after earthquake took place. About 23.9% of respondents had access to disposable sanitary pads for managing the menstrual bleeding. Of these 28 respondents, 42.8% had borrowed them either from a relative or friend, 39.3% bought from vendors, and 17.9% used the remaining pads from a previous occasion. Almost 76% of respondents used reusable cloths for managing menstrual bleeding, made mostly from old clothes (78.7%). About 85% of respondents who could use disposable sanitary pads disposed the used pads at a random nearby open and uninhabited space. Majority of respondents (77.8%) preferred disposable pads as adsorbents, as they were perceived to be cleaner, more hygienic, easier to use and more comfortable than alternative options. For those respondents who preferred reusable cloth as adsorbents (22.2%), easy availability, reusability, and habit were the reported reasons (Table 3).

**Table 3:** Menstrual Hygiene Management-related responses related to first month post-earthquake (*n* = 117)

Variables	*n* (%)
Received adsorbents as relief material	
Did not receive	117 (100.0%)
Receive soap as relief material	
Did not receive	59 (50.4%)
Received as relief material	58 (49.6%)
Privacy available for MHM practice	
Not adequately available	67 (57.3%)
Adequately available	50 (42.7%)
Timing to remember adsorbents as a need	
On the day of earthquake	15 (12.8%)
Only when menstruation occurred	102 (87.2%)
Type of adsorbents used after the earthquake	
Disposable Pads	28 (23.9%)
Reusable Cloth	89 (76.1%)
Source of the adsorbents? (Disposable pads; *n* = 28)	
Relative or Friend	12 (42.8%)
Bought	11 (39.3%)
Remaining from last time	5 (17.9%)
Source of the adsorbents? (Reusable cloths; *n* = 89)	
Old clothes	70 (78.7%)
Relative or Friend	19 (21.3%)
Disposal site for used disposable pad (*n* = 28)	
Along with other waste	4 (14.3%)
Separately in a nearby open space	24 (85.7%)
Preferred choice of adsorbents	
Disposable Pads	91 (77.8%)
Reusable Cloth	26 (22.2%)
Reason for preferring Disposable pads (*n* = 91)	
Clean	47 (40.2%)
Hygienic	18 (19.8%)
Easy to use	15 (16.5%)
Comfortable	11 (12.1%)
Reason for preferring Reusable cloth (*n* = 26)	
Easy availability	14 (53.8%)
Reusability	10 (38.5%)
Habit	2 (7.7%)
Perceived sustainable choice in disasters	
Disposable Pads	31 (26.5%)
Reusable Cloth	86 (73.5%)

Age, education, marital status and the type of adsorbents used before were associated with the type of adsorbents used during the menstruation immediately after the earthquake. In post-earthquake situation, women who are in the age group of 15-34 years used reusable sanitary cloth 3 times more (OR = 3.14; CI = (1.07-9.20) than the girls in the age group of 15-24 years. Women & girls who had no school education used reusable sanitary cloth 9 times more (OR = 9.68; CI = 2.16-43.33) than those who had some school education. Married women & girls used reusable sanitary cloth 3 times more (OR = 2.99; CI = 1.22-7.31) than those who were unmarried. Women & girls who used reusable sanitary cloth prior to earthquake used reusable sanitary cloth 5 times more (OR = 5.82; CI = 2.33-14.55) than those who used disposable pads prior to the earthquake (Table 4).

**Table 4:** Factors affecting the use of adsorbents in immediate post-earthquake Nepal

Variables	Type of Adsorbents used after earthquake		Unadjusted OR (95% CI)	*p*-value
	Reusable Cloth (%)	Disposable Pads (%)		
Age				
15-24 years	27 (64.3)	15 (35.7)	1	
25-34 years	34 (85.0)	6 (15.0)	3.14 (1.07 – 9.20)	0.031
35-45 years	28 (80.0)	7 (20.0)	2.22 (0.78 – 6.29)	0.128
Education				
School education	51 (66.2)	26 (33.8)	1	.000
No School Education	38 (95. 0)	2 (5.0)	9.68 (2.16 – 43.33)	
Marital Status				
Unmarried	20 (60.6)	13 (39.4)	1	.014
Married	69 (82.1)	15 (17.9)	2.99 (1.22 – 7.31)	
Type of Adsorbents used before Earthquake				
Disposable Pads	21 (53.8)	18 (46.2)	1	.000
Reusable Cloth	68 (87.2)	10 (12.8)	5.82 (2.33 – 14.55)	

### Qualitative findings

Experience of managing first menstrual period immediately after the earthquake was explored using qualitative technique. The in-depth interviews were coded into the three themes, *1. Experiences of women in accessing adsorbents, 2. Personal hygiene related issues and 3. Finding safe space during the first menstrual period*. Five women described in short in-depth interviews their experience of managing their first menstrual period after the earthquake. Even during a desperate situation such as the event faced by the respondents, menstrual hygiene management was still seen as a private issue. This belief also extended to where they went for help. As a collectivistic society [34], it was still possible for women to share problems with friends and get some help when available.

### Experiences of women in accessing adsorbents

The respondents said:

*“…sanitary pads did not strike my mind until four days after the earthquake during afternoon when my periods began. My brother asked me if there was a problem as I was staying inside for a long time. I just couldn’t say out loud that I started my periods. I stayed inside the tent until dark and then I started asking my close friends and I got some disposable pads, which I promised to return in a few days.” (15-19 years; high school graduate; respondent ID 1)*

*“I used disposable pads before, but I had to make do with my mother’s old cotton saree cloth, which came handy.” (25-29 years; university graduate; respondent ID 2)*

### Personal hygiene related issues

One woman reported that her menstrual period started in the evening after the earthquake. She could not tell her mother or other friends, as she felt that everyone was stressed and overwhelmed by the chaos surrounding the earthquake. She mentioned that:

*“I started to bleed on the same day…Only late at night, I was able to approach my mother. We managed to make a napkin from an old cotton saree. I only wore my napkin the following morning. There was no soap to take bath and I had to change before the sun came out.” (20-24 years; no schooling; respondent ID 4)*

### Finding safe space during the first menstrual period

Apart from the lack of adsorbents, privacy was highlighted as a serious issue. As an issue not openly discussed in society and pertaining just to women, respondents felt that only women would be able to relate to the suffering they had to endure as a result of the lack of resources and privacy:

*“I used reusable cloth before as well, so managing a cloth was easy. I found it quite difficult to locate a safe place where I could have some privacy even if it is was just for a few minutes. There were people everywhere.” (20-24 years; educated up to 8th grade; respondent ID 3)*

One of the respondents further elaborated on the lack of safe spaces with adequate privacy:

*“I used an old cloth during my menstrual periods. My neighbor found me a secluded area behind the trees where I was told that other women also changed their pads/cloth. I was horrified at the small pile of sanitary cloth disposed openly just a little distance away.” (35-39 years; no schooling; respondent ID 5)*

Field notes corresponded with the qualitative expression of the disposal of used pads. There was no designated space for waste disposal and used pads were disposed in open space nearby the place where women and girls went to change the pads.

## Discussion

This mixed methods study highlights the experiences of women and young girls related to menstrual hygiene management in the aftermath of the recent earthquake in Nepal. Although menstrual hygiene management needs are only specific to a subgroup of the population, they appear as an immediate pressing requisite for the women affected and as such, they should be seen as another core need even in post-disaster scenarios. The quantitate findings reveal that MHM as a priority that was not immediately addressed, however women and girls also list MHM material as less pressing compared to other general needs in immediate post-earthquake situation. The qualitative data presents evidence that for the menstruating women, it indeed was a challenge in timely accessing the material as well as availability of private space for the MHM activities. Our study reiterates the importance of MHM activities for women and adolescent girls and highlights the necessity to design culturally appropriate and sustainable interventions embedded within well-planned interventions in disaster relief efforts to support women and young girls.

Our findings indicate that whilst MHM activities are felt as important priorities for women and girls, they are often overlooked during disasters. Ensuring access to an essential amount of water available for personal use, provision of ready-made hygienic kits containing essential medicines, first aid and sanitary pads, and ensuring women’s safety and dignity in using toilets and wash areas are fundamental rights of every woman during disasters and must therefore be safeguarded.

While further participatory research methods could enrich the existing evidence-base, particularly around personal preferences and socio-cultural aspects of menstrual hygiene management, our use of in-depth interviews illuminated the findings from the quantitative data and facilitated its understanding.

In this study, most respondents did not receive any menstrual hygiene adsorbents in the relief materials during the first month post-earthquake. Hence, women had to improvise by making reusable sanitary towels from sarees or cloths, or obtain through their own networks of friends and relatives. While this seems to be the only option during emergencies, these adsorbents might not be hygienic and could potentially lead to infections [3, 18]. Only half of the respondents in this study received soaps as relief material in the first month following earthquake. Availability of a safe, private space for changing was not available for more than half of the respondents. Safe disposal of sanitary pads was also a concerning issue, echoing the findings reported among displaced girls and women in Myanmar and Lebanon, where some women disposed of used pads together with household waste in dark colored plastic bags [35]. Women and girls in India also report disposal of used sanitary pads in inappropriate places [16].

Our finding on the use of reusable sanitary towels is in concordance with practices reported by others in rural areas of Nepal [12]. In immediate post-disaster situations, most women and girls who menstruate during such events may perceive MHM activities as an immediate, peremptory need. Our findings corroborate with news reports from Nepal [27, 36] and from literature which indicate that menstrual hygiene management is often not prioritized in relief works [22]. Such low attention could also point to societal taboos and inadequate sensitivity on MHM within the wider society [9, 19, 37]. Research from elsewhere also supports that menstrual hygiene being exclusively a feminine need is lesser priority in humanitarian responses whereas, the food and security needs expressed by participants are the fundamental immediate needs for everyone [24]. The underlying reasons for the lack of adsorbents within standard relief materials supplied to earthquake victims and survivors on the first month would require further research among the humanitarian workers, logisticians and agencies.

Immediate measures during disasters are considered possible using locally available cloth as adsorbents [13]. The efforts need to emphasize the need for private spaces for women focusing on menstrual hygiene management apart from the toilets. Availability of a toilet still leaves an unmet need for addressing the women’s need for menstruation related privacy [38]. Safe disposal of sanitary pads is also essential [39]. The menstruating girl not being able to discuss the need for materials to manage her periods expressed in this study is an example of how silent the women and girls are about menstruation and related matters. The inability of women to freely talk about menstruation within their communities suggests that there are still taboos associated with menstruation [40]. In the south Asian context especially in India and Nepal, menstruation comes with many social restrictions including for religious rituals [9, 12, 38].

Lessons from Myanmar and Lebanon suggests that, women and girls’ choice of adsorbents in emergency settings depend on the availability of the materials, privacy for changing the pads and availability of private space for washing and drying the reusable towels or disposing of pads [35]. Most respondents in our study referred using reusable instead of disposable sanitary pads, as these were unavailable despite being more convenient, a predicament also reported by authors in other settings [35]. Reusable sanitary towels seem to be more easily available, and are perhaps a sustainable and culturally suitable alternative in the Nepalese context.

The current study has certain limitations. It was conducted in one earthquake-affected district of Nepal and may not therefore be generalizable to all other areas of Nepal. As our study setting was predominantly rural, our findings may be particularly unsuitable to urban areas of the country. Further, we specifically asked questions related to the first menstrual period management after the earthquake, and as the study was conducted 3 months after the earthquake potential recall bias cannot be overlooked.

## Conclusion

The study brings evidence that MHM activities are lesser prioritized in immediate relief works. As menstruation comes along with cultural and religious taboo, the MHM needs is present as a silent need, which is addressed by the use of local materials and resources in immediate post-disaster situations. The choice of materials for adsorbents is made based on the availability of materials and the privacy for MHM activities While disposable pads are considered more convenient and hygienic, the use of culturally appropriate, local materials and gender sensitive methods of reusable sanitary towels could be considered by relief workers as sustainable and environmental friendly method.

## Abbreviations

MHM: Menstrual hygiene management
